# The tumor microenvironment of hepatocellular carcinoma and its targeting strategy by CAR-T cell immunotherapy

**DOI:** 10.3389/fendo.2022.918869

**Published:** 2022-08-25

**Authors:** Zhang Guizhen, Ji Guanchang, Liu Liwen, Wang Huifen, Ren Zhigang, Sun Ranran, Yu Zujiang

**Affiliations:** ^1^ Department of Infectious Diseases, The First Affiliated Hospital of Zhengzhou University, Zhengzhou, China; ^2^ Presion Medicine Cencter Gene Hospital of Henan Province, Zhengzhou, China; ^3^ Academy of Medical Sciences, Zhengzhou University, Zhengzhou, China; ^4^ Department of Urology People’s Hospital of Puyang, Puyang, China

**Keywords:** hepatocellular carcinoma, tumor microenvironment, immunotherapy, adoptive cell therapy, chimeric antigen receptor

## Abstract

Hepatocellular carcinoma (HCC) is the major subtype of liver cancer, which ranks sixth in cancer incidence and third in mortality. Although great strides have been made in novel therapy for HCC, such as immunotherapy, the prognosis remains less than satisfactory. Increasing evidence demonstrates that the tumor immune microenvironment (TME) exerts a significant role in the evolution of HCC and has a non-negligible impact on the efficacy of HCC treatment. In the past two decades, the success in hematological malignancies made by chimeric antigen receptor-modified T (CAR-T) cell therapy leveraging it holds great promise for cancer treatment. However, in the face of a hostile TME in solid tumors like HCC, the efficacy of CAR-T cells will be greatly compromised. Here, we provide an overview of TME features in HCC, discuss recent advances and challenges of CAR-T immunotherapy in HCC.

## Introduction

Primary liver cancer, represents the sixth most commonly diagnosed cancer and the third leading cause of cancer-related mortality currently according the Global Cancer Statistics 2020, with approximately 906,000 new cases and 830,000 deaths (1). In the vast majority of cases, HCC frequently develops from cirrhosis, caused by viral (hepatitis B or C virus) and non-viral (alcoholic or non-alcoholic fatty liver disease) risk factors (2). Frustratingly, HCC is an insidious tumor often diagnosed in advanced stage. For the patients with advanced stages, the treatments of choice are usually palliative. Despite aggressive treatment regimes, including surgery, combined radio and chemotherapy, HCC patients will still experience tumor recurrence and metastasis with the death rates increasing by 2–3% per year (3, 4). Therefore, identification new factors underlying therapy resistance and novel therapeutic strategies for HCC are urgently needed.

Among patients with HCC who are diagnosed as the same TNM stage and experience similar clinical management, clinical outcomes are different, indicating that HCC is highly heterogeneous. Additionally, the complexity of heterogeneity is not only reflected in different patients, but also reflected in the disease progression and treatment courses of the individual patient (5). Recent accumulating evidence has revealed that this extraordinarily heterogeneity is closely related to TME of HCC, and contributes to the inconsistent outcome of anti-cancer therapy. Consequently, TME received considerable attention in recent years, and targeting TME is increasingly recognized as a new battlefield for HCC therapy, especially immunotherapy including vaccines, antibodies, immune checkpoint inhibitors, and adoptive cell therapy (ACT), such as CAR-T cells (6–8). CAR-T cell therapy, as the most encouraged immunotherapy, has made great strides in hematological malignancies. Meanwhile, intensive endeavors to target HCC by CAR-T has demonstrated promising efficacy with manageable toxicity and safety. The present review aims to provide a comprehensive picture of TME in HCC, discuss efforts to develop treatments by CAR-T.

## Overview of TME in HCC

**Figure 1:**
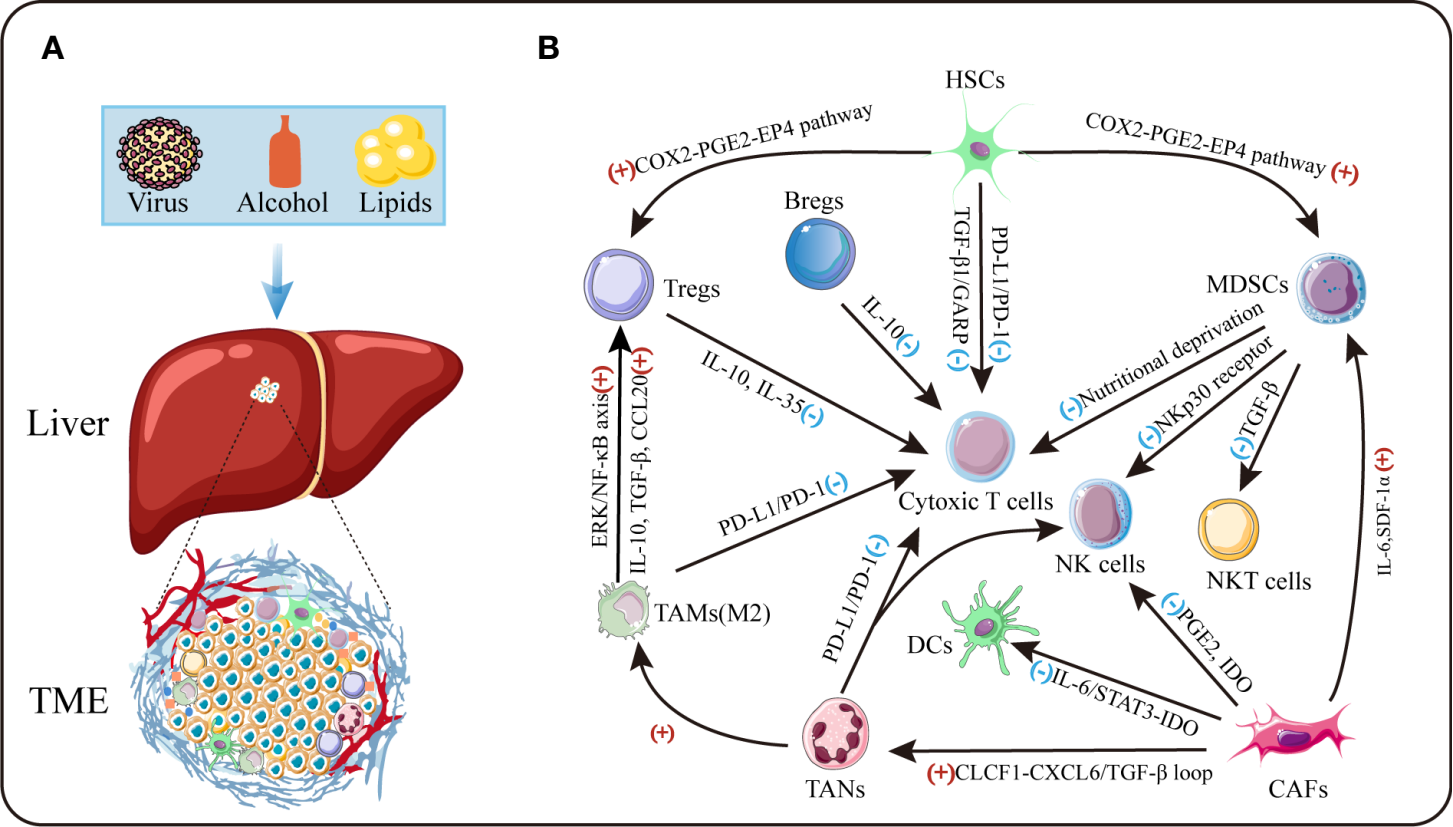
The tumor microenvironment of HCC. **(A)** TME is the cellular milieu in which the HCC cells grow. **(B)** Crosstalk among diverse suppressive immune cells in TME. IDO:indoleamine 2,3-dioxgenase.

The tumor microenvironment is an intricate system, which comprises cellular and non-cellular components (**Figure 1**). The major cellular components include tumor cells, activated hepatic stellate cells, myeloid-derived suppressor cells (MDSCs), cancer-associated fibroblasts (CAFs), tumor-associated macrophages (TAMs), tumor-associated neutrophils (TANs), immune and endothelial cells (9, 10). Produced by these cells, the tumor stroma includes extracellular matrix (ECM) proteins, proteolytic enzymes, cytokines and growth factors (7). Crosstalk between cancer cells and TME has been identified to have a profound effect on cancer progression through prompting cell proliferation, survival and the ability of migration and evasion. Thus, a better understanding of the adverse TME would facilitate to develop novel therapeutic approaches for treatment of HCC in future.

### Hepatic stellate cells (HSCs)

HSCs are the most principal cell players responsible for collagen synthesis in the liver and have a quiescent and an activated state, the latter being transformed from the former upon liver injury (11). Activated HSCs (a-HSCs) can produce the extensive accumulation of ECM in chronically damaged livers, leading to the development of hepatic fibrosis (12).

Although some researchers advocate that HSCs act as a tumor suppressor in HCC, the mainstream view is that HSCs in TME may facilitate tumor growth, involving tumor angiogenesis, invasion and metastasis (13). It was reported that a-HSCs strongly affect the malignant phenotype of HCC *via* paracrine feedback mechanisms through activating NF-κB and extracellular regulated protein kinases (ERK), two major signaling pathways in hepatocarcinogenesis (14). Another research demonstrated that HSCs can be activated under acidic condition depending on the phosphorylation of ERK1/2 and secrete osteopontin to promote HCC metastasis (15). Also, IL-6/STAT3 pathway has proven important, by which HSCs increased cancer cell viability and migration ability in HCC (16). Furthermore, Franziska and colleagues have identified that proteinase-activated receptor 2 expressed by HSCs can promote secretion and migration of pro-mitotic and pro-angiogenic factors to accelerate HCC growth (17). HSCs’ function in angiogenesis was also verified in Lin’s research (18). As elucidated by Yuta et al, in the HCC microenvironment, an increase of HSCs may be involved in tumor progression by producing GDF15 in an autophagy-dependent manner (19). In addition, mechanistic studies indicated that a-HSCs can accelerate HCC progression through miR-1246-RORα-Wnt/β-catenin axis (20). Furthermore, HSCs can indirectly affect HCC by cross talking with immune cells and impairing immune surveillance. A-HSCs have been elucidated to aggravate HCC by interacting with monocytes and macrophages, shifting them from an inflammatory to an immunosuppressive phenotype (21, 22). Li et al. provided the evidence that HSCs inhibit T cells proliferation and IFN-γ production through active TGF-β1 from a cell-surface-bound latent TGF-β1/GARP complex (23). It could also act in an autocrine fashion for HSCs to indirectly induce T cells apoptosis through upregulating expression of programmed death-ligand 1 (PD-L1) (24). Notably, HSCs can also induce regulatory T cells (Tregs) and MDSCs probably through activating COX2-PGE2-EP4 pathway provide an immunosuppressive milieu for HCC (25, 26). Clinically, it’s well evidenced that HSCs are associated with recurrence and poor survival of patients with HCC (21, 27). Collectively, previous studies unraveled the significant role of HSCs in HCC progression and presented possibilities for HSCs as therapeutic targets.

### Myeloid-derived suppressor cells (MDSCs)

MDSCs, characterized by a pathological state of activation, represent a heterogeneous population of immature myeloid cells, and exert inhibitory function in antitumor immunity in patients (28, 29). Studies investigated that CXCL1/CXCR2 and CCL26/CX3CR1 axis are two important pathways that induce the homing of MDSCs to the HCC microenvironment, thereby promoting immune escape and tumor growth (30, 31). Additionally, HIF-1α exerts a critical role by recruiting MDSCs into the hypoxia region of HCC foci *via* mediating ENTPD2 over-expression in HCC cells (32).

As a powerful inhibitory immune modulator, infiltrated MDSCs exert versatile immunosuppressive effects in HCC by inhibiting effector T cells, reducing natural killer (NK) cells cytotoxicity, expanding immune checkpoint signaling through diverse mechanisms. Researchers have reported that MDSCs suppressed autologous T cell proliferation and activation by depleting energy resources (e.g. arginine and cysteine) (33). Interestingly, Baumann et al. identified that T cells can be stunned by MDSCs *via* cell-cell transfer of the metabolite methylglyoxal (34). Infiltration into tumor sites is a prerequisite for immune cells to exert anti-tumor effects. Unfortunately, it was reported that MDSCs are significantly associated with reduced tumor infiltrating lymphocytes (TILs) in HCC (35). Additionally, MDSCs can reduce cytotoxicity and cytokine release of NK cells via the NKp30 receptor (36). Mechanistic studies indicated NKT cells are also one of the targets of MDSCs to exert immunosuppressive effects by selectively suppressing the secretion of IFN-γ deriving from NKT cells (37). As such, MDSCs can allow tumor cells to evade immune surveillance by interacting with other immune cells. Evidence has shown that MDSCs promote tumor growth and are associated with diminished efficacy of immunotherapy (38, 39).

### Cancer-associated fibroblasts (CAFs)

CAFs are defined as the fibroblastic type of cells in a tumor mass, which are thought to interplay tightly with cancer cells (40). As an abundant and active cell type within the TME, CAFs are mainly activated from resident fibroblasts, stellate cells, mesenchymal stem cells or mesothelial cells, but evidence from a lineage-tracing analysis is still lacking (40, 41).

Although there is no denial that CAFs may exert a tumor-suppressing function, recent emerging data has convincingly indicated the tumor-promoting effects of CAFs. In tumors, CAFs function as remodeling machine to aid the creation of a desmoplastic TME and the signaling center to participate in the crosstalk with tumor and non-tumor cells (41). Firstly, CAFs could facilitate HCC cells epithelial-to-mesenchymal transition (EMT) through the transglutaminase 2-dependent IL-6/IL6R/STAT3 pathway, and promote HCC metastasis by activating HIF1α/ZEB1 axis (42–44). Secondly, a great deal of findings reported that CAFs could accelerate tumor growth by producing epidermal growth factor (EGF), fibroblast growth factor (FGF), hepatocyte growth factor (HGF), cytokines and chemokines (45–47). An *in vitro* experiment demonstrated that CAFs activated by TIMP-1 markedly inhibited HCC apoptosis by upregulating BCL-2/BAX ratio *via* SDF-1/CXCR4 axis (48). Also, Mano et al. provided important evidence that endogenous and exogenous BMP4 play a key role in the transformation of fibroblasts to CAFs which subsequently produce large amounts of cytokines to enhance invasiveness of HCC cells (49). Notably, CAFs-mediated cellular crosstalk is another important mechanism by which they promote tumor progression. Very recently, a study revealed that CAFs-derived CLCF1 could increase the secretion of CXCL6 and TGF-β in HCC cells, which subsequently enhance stemness of cancer cells and promote TANs infiltration and polarization in autocrine and paracrine manners, respectively. Interestingly, CXCL6 and TGF-β in turn activate CAFs to express more CLCF1, thus forming a positive feedback loop that promotes tumor progression (50). Moreover, it’s well evidenced that IL-6 and SDF-1α derived from CAFs can induce MDSCs generation, which subsequently impairs T-cell proliferation and alter the phenotype and function of T cells, which create favorable conditions for HCC progression (51). Crosstalk between CAFs and other cells such as NK and dendritic cells was also reported (52, 53).

### Tumor-associated macrophages (TAMs)

TAMs are termed as macrophages within the tumor stroma and play pro-tumoral or sometimes anti-tumoral roles due to the ability to acquire M1 (classic) or M2 (alternative) phenotype-depending on signals from the tumor stroma (54). The classically activated macrophages or M1-type which exert their cytotoxic function through their T cell-stimulating activity, can be induced by Th1 cytokine such as INF-γ and through Toll-like receptor 4 engagement. Unfortunately, TAMs are also polarized towards an M2 phenotype with decreased antigen-presenting ability by Th2 cytokines IL-4/IL-13, functioning immunosuppressor in the TME (54, 55).

An immunogenic analysis showed that macrophages are prone to polarize to the M2 phenotype in HCC. Patients with high presence of M2 macrophages tend to have a more aggressive phenotype (56). A great deal number of studies confirmed and extended this observation. Bartneck’s study demonstrated that immunosuppressive TAMs are abundant in the center of HCC and that CCR2^+^ TAMs accumulate at the highly vascularized border of tumor; *In vivo* experiments showed that inflammatory and angiogenic pathways are activated in CCR2^+^ TAMs (57). Consistently, TAMs accumulation had significant prognosis value in HCC patients (56). Mechanistically, TAMs can produce cytokines such as VEGF, EGF, platelet-derived growth factor (PDGF) to promote tumor angiogenesis, and matrix metalloproteinases secreted by TAMs can remodel TME to facilitate tumor metastasis (58). In addition, TAMs can also induce Treg cells infiltration into tumor tissue *via* producing cytokines and chemokines, such as IL-10, TGF-β and CCL20 (59, 60). As elucidated by Wu and colleagues, TREM-1^+^ TAMs promote the recruitment of CCR6^+^Foxp3^+^ Tregs through the ERK/NF-κB axis, which endows HCC with anti-PD-L1 therapy resistance (61). Kupffer cells, which are liver-resident macrophages,can inhibit CD8^+^ T cytotoxicity by PD-L1/PD-1 interaction and thus inhibit CD8^+^ T-dependent immune response (62). Hence, the role of TAMs in HCC deserves much attention and TAMs may be a promising target in the treatment of HCC. It has been verified that macrophages mediate sorafenib resistance in HCC and TAMs depletion can improve the therapeutical efficacy of sorafenib (63, 64).

### Tumor-associated neutrophils (TANs)

Neutrophils, derived from the bone marrow, are the first subset of immune cells to be recruited to lesions responding against infectious and inflammatory insults (65). In TME, neutrophils infiltrating into lesions can exhibit N1 (anti-tumoral) or N2 (pro-tumoral) phenotype-depending on the presence of TGF-β (66, 67).

As one of the most abundant components in HCC, Neutrophils have been recognized to play pivotal roles in regulating cancer development. It was verified that increased intra-tumoral neutrophils are correlated strongly with decreased recurrence free survival (RFS)/overall survival (OS) and can act as an independent prognostic factor in HCC patients (68). These results were reinforced in other studies (69–71). Zheng’s and his coworkers provided evidence that neutrophils can be induced by IL-17 to migrate to tumor stroma through epithelial cell-derived CXC chemokines; Besides, high infiltration of TANs is positively associated with angiogenesis at tumor-invading border of HCC (72). They further identified that TANs also perform autophagy *via* the synergy of ERK1/2, p38 and NF-κB signaling axis and subsequently facilitate tumor progression by enhancing the secretion of OSM and MMP9, suggesting a regulatory loop between tumor cells and neutrophils (73). A positive feedback loop was also verified and exerts an essential function in the generation of stem-like cells in HCC (74). In addition, as the major source of c-Met ligand HGF, the accumulated neutrophils can actively promote the metastasis of HCC through the HGF/c-Met pathway. Of note, high infiltration of neutrophils in HCC determined malignant cell c-Met-associated clinical outcome of patients (75). A series of studies have shown that TANs also interact with other immune cells to exert their tumor-promoting function. The research conducted by He and colleagues has unraveled that infiltrating neutrophils express a higher frequency of PD-L1 in the presence of GM-CSF and TNF-α in TME; In turn, the PD-L1^+^ neutrophils effectively impaired anti-tumor immunity *via* suppressing the proliferation and activation of T cells through the PD-L1/PD-1 signaling axis (76). Another study came to a similar conclusion (77). These TANs could also drive HCC progression and sorafenib resistance by recruiting macrophages, Treg cells and NK cells (69, 78).

### Lymphocytes

Regulatory T cells (Tregs), defined as CD4^+^ and CD8^+^ T cells with immunosuppressive function, are known for their critical role in suppressing inflammation, and thus can antagonize the anti-tumor effect of immune responses (79). Studies have shown that Tregs are the main type of tumor-infiltrating T cells in HCC, which can significantly prejudice CD8^+^ T cells proliferation, activation and suppress cytolytic molecule release and production of CD8^+^ T cells like granzymes, perforin (80). It was also evidenced that Tregs promote HCC invasion *via* TGF-β1-induced EMT (81). Tregs mediate sorafenib resistance, and blocking Tregs with inhibitors can overcome sorafenib resistance and increase tumor sensitivity to immunotherapy (82).

Recently, a research demonstrated that HCC tissue has a significantly higher TIM-1^+^ regulatory B cells (Bregs) infiltration than the adjacent benign tissue. These Bregs show a CD5^high^CD24^-^CD27^-/+^CD38^+/-^ phenotype, secrete much immunosuppressive cytokine IL-10 and suppress CD8^+^ T cells strongly. In addition, the infiltration of Bregs is correlated with advanced disease stage, predicted early recurrence and decreased survival of patients with HCC (83). It was verified that CD40/CD154 signaling axis may be one of the pathways by which Bregs promote HCC progression (84).

Th17 cells are CD4^+^ lymphocytes producing IL-17. Wang’s work demonstrated that Th17 synchronically increases with Tregs and Bregs in the peripheral circulation and in tumor tissues in HCC patients. Also, further studies indicated that the production and proliferation of Th17 are promoted by tumor cells in TME mainly through cell-contact independent mechanisms (85). Furthermore, clinical studies showed that increased Th17 cells in tumor stroma are correlated with poor survival and higher postoperative recurrence, suggesting that Th17 cells may facilitate the development of HCC (86).

### Tumor-associated endothelial cells (TAECs)

Endothelial cells are essential components in the process of tissue vascularization. It has been verified that migration of endothelial cells to tumor sites promotes the formation of the tumor neo-vasculature. TAECs express angiogenic surface receptors, such as VEGFR, EGFR and CXCL12, which conduct signaling through the interaction with their corresponding ligands to regulate endothelial cell survival, proliferation, mobilization, and invasion (87, 88). Compared with those in normal tissues, TAECs have accelerated cell cycle, increased ability of migration, and overexpressed CD105 and TGF-β1. TGF-β1 promotes the recruitment of CD105^+^ endothelial cells, thereby contributing to angiogenesis of tumor (89). CD105^+^ endothelial cells could make HCC resistant to chemotherapeutic drugs and angiogenesis inhibitors by increasing angiogenic activity of tumors (90).

### Extracellular matrix

ECM, consisted of proteoglycans, glycoproteins and hyaluronan, is one of the major components of tumors that exert various crucial functions, including structural support, modulation of the microenvironment, and mediating intercellular communication (91). As one of the major components of TME, dysregulation of the ECM is a distinctive feature in cancer (91, 92).

Heparin sulfate, chondroitin sulfate, and keratan sulfate are the major components of proteoglycans in ECM, providing binding receptors for growth factors, cytokines, chemokines and are involved in many physiological and pathological processes (91). For example, Heparin sulfate proteoglycan (HSPG) acts as a co-receptor for binding of FGF-2 to its cognate FGF receptors, thus forming a ternary complex critical for cell proliferation and angiogenesis (93, 94). Glypican 3 (GPC3), an oncofetal HSPG anchored to the cell membrane, exhibits elevated expression in tumor cells and tumor vascular cells in HCC, and its expression correlates with a poor prognosis (95). Mechanistically, the oncogenic role of GPC3 is exerted through Wnt/β-Catenin pathway (96). SULF1 and SULF2 are two heparin-degrading endosulfatase enzymes that regulate heparin-dependent signaling in cells by altering the sulfation of HSPGs. Decreased expression of SULF1 was verified in the majority of HCC cell lines and approximately 30% of HCCs, but almost all HCC cell lines and 60% of HCC samples demonstrate elevated expression of SULF2 (97). Besides, upregulation of SULF2 predict a significant poor prognosis and higher postoperative recurrence rates (98, 99). Mechanistically, Lai et al. found that overexpression of SULF2 could enhance GPC3 expression and exert the oncogenic role by GPC3-dependent Wnt activation (99).

Collagens are the most abundant ECM proteins to support mechanical structure. Aberrant expression of collagen probably acts as a barrier for tumor metastasis, but may also promote tumor metastasis as a foothold for its movement (100). It was verified that COL1A1 is highly expressed in HCC tumor tissues compared with benign tissues and confers a survival advantage to liver cancer cells and enhances their oncogenicity (101).

Laminin, together with collagen, constitutes a component of the basement membrane. Laminin is involved in various biological activities, including cell adhesion, growth, differentiation, migration and angiogenesis (100). Laminin-5, which is not detected in normal liver, but overexpressed in HCC tissues, correlating with more proliferative and metastatic phenotypes. Together with TGF-β, Laminin-5 promotes EMT by upregulating Snail and downregulating E-cadherin in HCC (102). Also, Laminin B1 stimulates integrin-dependent focal adhesion kinase/Src proto-oncogene non-receptor tyrosine kinase signaling and supports tumor progression at the invasive front of HCC through the PDGFRα-laminin B1-keratin 19 cascade (103).

Taken together, these data provide solid evidence supporting the important role of the TME in the development of HCC and reveal the complex interaction among the components in the TME, also explain why traditional therapy fail in the treatment of HCC, and thus the development of novel therapeutic modalities is urgently needed.

## Chimeric antigen receptor T cell immunotherapy

**Figure 2:**
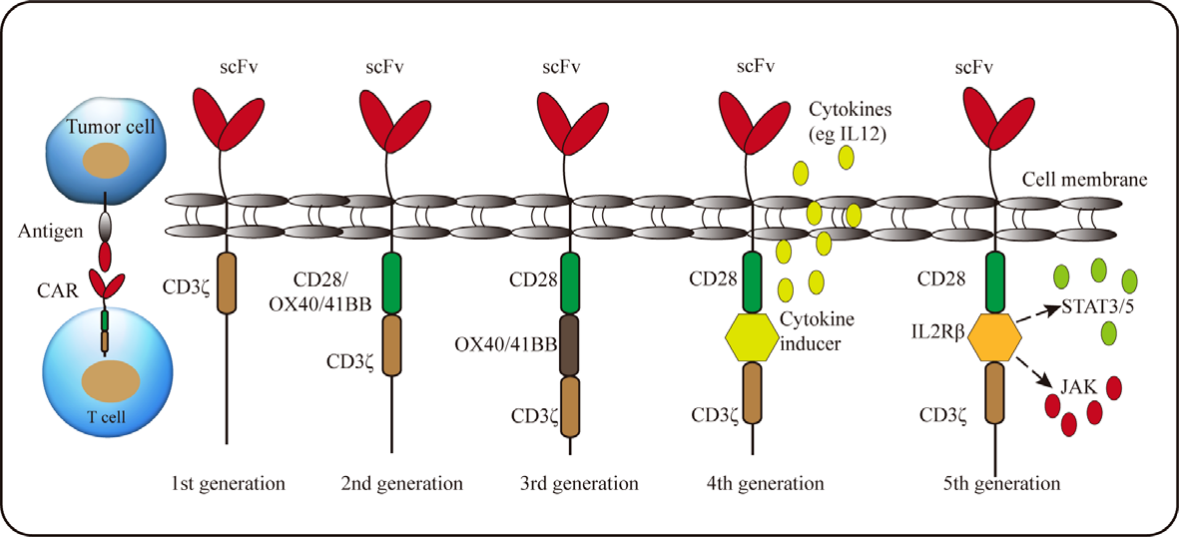
Schematic representation of the CAR structure. A CAR consists of single-chain variable fragment with a hinge, transmembrane domain, and CD3ζ (1st generation). 2nd generation and 3rd generation CARs contain one or two costimulatory molecules, respectively. Whereas the signal domain of the 4th generation CAR includes an inducible cytokine cassette. The 5th generation CAR encodes a truncated cytoplasmic domain of IL-2 receptor β with a binding site for the STAT3 to activate JAK-STAT pathway.

A recently-developed adoptive cell therapy is to generate tumor-specific CAR-T cells. The typical CAR is composed of an extracellular single-chain variable fragment (scFv) for recognizing antigens, a hinge region to provide flexibility, a transmembrane region and intracellular signaling domain (104). Due to the requirements of functional improvement, the intracellular signaling domain can be modified, and according to this, CAR can be divided into five generations (**Figure 2**). The first generation CARs only have a single signal domain CD3ζ chain for T-cell activation; The second and third generation CARs are characterized by the addition of one or two costimulatory domains, respectively; Most commonly derived from CD28 or 4-1BB, costimulatory domains endow CAR-T cells improved proliferation and persistence, enhanced cytokine secretion and increased anti-tumor cytotoxicity; The fourth generation CAR-T incorporates a costimulatory domain and inducible cytokine cassette and is termed “T cells redirected for universal cytokine-mediated killing” (TRUCK) to release the proinflammatory cytokines to activate innate immune response against the tumor and resist inhibitory components in TME, such as Tregs and MDSCs (104, 105). Recently, the fifth generation CARs, encoding a truncated cytoplasmic domain from IL-2Rβ and a STAT3-binding tyrosine-X-X-glutamine motif, together with CD3ζ and CD28 domains, were also developed. These novel CARs can activate the JAK-STAT signaling pathways in an antigen-dependent manner, which confers CAR-T cells superior persistence and antitumor effect (106).

Compared with the traditional T cell receptor-T cells, CAR-T can specifically recognize a wide array of antigens in a non-major histocompatibility complex (MHC) restricted manner, and solve the immune escape caused by the downregulation of MHC molecules (107). Furthermore, additional genes could be introduced to modify intracellular signaling domains enabling T cells resistant to immune suppression. CAR-T therapy has shown remarkable success for hematological malignancies and received its approval by the U.S. Food and Drug Administration as gene therapy which paves the way for further extension of this approach to solid malignancies including HCC (108, 109). In present, significant progresses have been made in the preclinical models and clinical trials utilizing CAR-T cells in HCC. Next, this review discusses several CAR-T targeting different antigens currently being evaluated in HCC. The latest clinical trials on CAR-T therapy for HCC is summarized in **Table 1**.

**Table 1:** Clinical trials in HCC using CAR-T.

NCT Number	Phase	Antigen	CAR-T Type	Status		Sample Size (n)	Conditions	Outcome Measures
NCT05123209	I	GPC3	2nd generation Autologous	Recruiting	12		LC	1 AEs 2 ORR, DOR, PFS and OS 3 Plasma α-AFP cells infusion 4 Persistence of CAR-T
NCT02932956	I	GPC3	N/A	Recruiting	10		LC	1 DLT 2 CR or PR 3 Median T cell persistence
NCT04093648	I	GPC3	2nd generation Autologous	Withdrawn	N/A		HCC	1 DLT 2 Response rate
NCT05003895	I	GPC3	N/A	Recruiting	38		HCC	1 Safety and feasibility 2 OS 3 Best overall response rate
NCT04951141	I	GPC3	N/A	Recruiting	10		HCC	1 AEs 2 ORR 3 OS
NCT04377932	I	GPC3	N/A Autologous	Recruiting	24		Basket including LC	1 DLT 2 Median CAR-T cell persistence 3 Best response as either CR or PR
NCT04715191	I	GPC3	N/A Autologous	Not yet recruiting	24		Basket including LC	1 DLT 2 Median CAR-T cell persistence 3 Best response as either CR or PR
NCT05103631	I	GPC3	N/A Autologous	Recruiting	27		LC	1 DLT 2 Median CAR-T cell persistence 3 Best response as either CR or PR
NCT02959151	I/II	GPC3	N/A Autologous /Donated	Unknown	20		Basket including HCC	1 AEs 2 Tumor response 3 Detection of CAR-T in the circulation
NCT03198546	I	GPC3	3rd/4th generation Autologous	Recruiting	30		HCC	1 DLT 2 Median CAR-T cell persistence 3 Best response as either CR or PR
NCT04506983	I	GPC3	2nd generation Autologous	Suspended	12		HCC	1 AEs 2 ORR 3 Proliferation ratio of CAR-T cells
NCT04121273	I	GPC3	2nd generation Autologous	Recruiting	20		HCC	1 DLT 2 Evaluation of tumor size 3 Peripheral tumor marker 4 Number of peripheral CAR-T cell
NCT03884751	I	GPC3	2nd generation Autologous	Completed	9		Advanced HCC	1 DLT, MTD and AEs 2 CAR-T expansion and persistence 3 PFS, ORR, OS, DOR, DCR and DOC
NCT02715362	I/II	GPC3	2nd generation Autologous	Unknown	30		HCC	1 AEs 2 Tumor response 3 Detection of CAR-T in blood 4 Serum cytokine levels
NCT02395250	I	GPC3	2nd generation Autologous	Completed	13		HCC	AEs
NCT02905188	I	GPC3	2nd generation Autologous	Active, not recruiting	9		HCC	1 DLT 2 Best response as either CR or PR 3 Median CAR-T cell persistence
NCT03980288	I	GPC3	4th generation Autologous	Completed	6		Advanced HCC	1 DLT, MTD and AEs 2 CAR-T expansion 3 ORR, DCR, DOC, DOR, PFS and OS
NCT03130712	I/II	GPC3	2nd generation Autologous	Unknown	10		Basket including HCC	1 AEs 2 Tumor response 3 Serum cytokine levels
NCT05155189	I	GPC3	2nd generation Autologous	Recruiting	20		HCC	1 AEs and limiting toxicities 2 Tumor response 3 Serum cytokine levels
NCT03146234	N/A	GPC3	N/A Autologous	Completed	7		HCC	1 Safety and tolerance 2 Engraftment 3 ORR, PFS and OS 4 Time of tumor progression
NCT05070156	I	GPC3	N/A Autologous	Recruiting	3		Advanced HCC	1 AEs, cellular kinetics 2 PFS, OS, ORR, DCR, DOR and DOC 3 Serum free GPC3, cytokines, CRP and lymphocyte subsets
NCT03084380	I/II	GPC3	2nd generation Autologous	Unknown	20		HCC	1 AEs 2 Overall complete remission rate 3 Duration of CAR-T in circulation
NCT05344664	I	GPC3	N/A	Not yet recruiting	12		HCC	AEs
NCT02729493	II	EpCAM	N/A Autologous	Unknown	25		LC	DCR
NCT03013712	I/II	EpCAM	3rd generation Autologous	Unknown	60		Basket including HCC	1 Toxicity profile 2 Persistence of CAR-T 3 Anti-tumor efficacy
NCT03672305	I	c-Met/PD-L1	N/A Autologous	Unknown	50		HCC	1 The efficacy of CAR-T in the treatment of HCC 2 AEs 3 The amplification and persistence of CAR-T
NCT05028933	I	EpCAM	N/A Autologous	Recruiting	48		Basket including HCC	1 DLT, MTD and AEs 2 ORR, DCR, DOR, PFS, OS 3 Level of tumor cells in peripheral blood
NCT04348643	I/II	CEA	N/A	Recruiting	40		Basket including LC	1 AEs 2 Persistence of CAR-T 3 ORR, DOR, PFS and OS 4 Levels of CEA, IL-6 and CRP in Serum
NCT03993743	I	CD147	3rd generation Autologous	Recruiting	34		Advanced HCC	1 DLT, MTD and AEs 2 Activity of CAR-T cell 3 CAR-T detection in extrahepatic sites
NCT02541370	I/II	CD133	N/A Autologous	Completed	20		Basket including LC	1 Occurrence of study related AEs 2 Anti-tumor responses to CAR-T
NCT03349255	I	AFP	2nd generation Autologous	Terminated	3		Basket including HCC	1 DLT 2 Response rate 3 CAR-T cell engraftment
NCT04550663	I	NKG2D	N/A Autologous	Not yet recruiting	10		Basket including HCC	1 MTD and AEs 2 Monitoring 3 ORR, PFS and OS

N/A, not available; AEs, adverse events; DLT, dose limiting toxicity; MTD, maximum tolerated dose; ORR, objective remission rate; CR, complete remission; PR, partial remission; PFS, progression-free survival; OS, overall survival; DOR, duration of response; DCR, disease control rate; DOC, duration of disease control. For more information, please visit the website: https://clinicaltrials.gov/.

### GPC3

Gao and his coworkers firstly reported the experience on CAR-T cells for the treatment of HCC. They constructed the first (αGPC3-Z CAR-T) and third generation CAR (αGPC3-28BBZ CAR-T) targeting GPC3. Results indicated that both generations of CAR-T specifically lysed HCC cell lines *in vitro*. αGPC3-28BBZ CAR -T cells could inhibit the growth of tumor in immunodeficient mice. It is of note that the third generation CAR-T cells secreted more IL-2 and IFN-γ, which has a positive correlation with the level of GPC3 expression on HCC cells (110). Another group reached the similar conclusion that the second and third generation CAR-T had a superior performance than the first generation construct *in vivo* (111). They found that T cells signaling *via* CD28 had higher cytotoxicity than those *via* 4-1BB *in vitro*; However, CAR-T cells containing the 4-1BB costimulatory domain had better proliferative activity *in vitro* and *in vivo*, indicating that the choice of costimulatory domain might affect the behavior of CAR-T cells. In two phase I trials, 13 patients with advanced HCC received autologous GPC3 CAR-T treatment to assess the safety. Most patients experienced manageable side effects, including pyrexia, decreased lymphocyte count, and grade 1/2 cytokine release syndrome (CRS). Grade 5 CRS occurred in only one patient and none of the patients experienced grade 3/4 neurotoxicity. The OS rates at 3 years, 1 year and 6 months were 10.5%, 42.0% and 50.3%, respectively. Additionally, two partial responses (PR) were confirmed. One patient with sustained stable disease (SD) was alive after 44.2 months (112).

In order to further improve the therapeutic efficacy, Pang et al. developed CAR-T cells which express IL-7 to induce proliferation and CCL19 to enhance migration of CAR-T cells. Results showed that incorporation of IL-7 and CCL19 into CAR-T cells remarkably promoted the antitumor ability. Surprisingly, these CAR-T cells eliminated the tumor completely 30 days after intratumor injection in a patient with advanced GPC3^+^ HCC in a phase I clinical trial (NCT03198546) (113). Similarly, another group verified that pretreatment the tumor by a recombinant adeno-associated virus carrying the CCL19 gene (AAV-CCL19) could increase the infiltration of GPC3 CAR-T to tumor tissue and significantly prolonged the survival time of mice (114). Besides, the impact of serum GPC3(sGPC3) on CAR-T treatment is also noteworthy. sGPC3 was reported to be associated with poor prognosis in postoperative patients with HCC (115). sGPC3 can competitively bind to CARs with membrane GPC3, but fail to activate CAR-T cells effectively, thus resulting in an inhibitory effect on CAR-T cells in HCC (116). Combination chemotherapy or immune checkpoint inhibitors may provide more possibilities for GPC3 CAR-T in the treatment of HCC.

### CD133

Expressed by cancer stem cells, CD133 is a pentaspan transmembrane glycoprotein. CD133 has attracted considerable attention as a potential cancer therapeutic target. Wang et al. constructed CD133-specific CAR-T cells (CD133 CAR-T) and found that CD133 CAR-T displayed distinctive lysis activity and secreted high level of cytokines targeting CD133^+^ cells and remarkably suppressed tumor growth *in vivo*. Surprisingly, high level of CAR gene copy was detectable in tumor tissue (117). Given these surprising results, they conducted a clinical trial (NCT02541370) to evaluate the antitumor effect of CD133 CAR-T cells in patients with advanced HCC. 21 patients were included and received CD133 CAR-T cells across phases I and II. Hyperbilirubinemia as the most common high-grade adverse event, this trial showed feasibility and controllable toxicities. The median OS and progression free survival (PFS) were 12 months and 6.8 months, respectively. Of 21 evaluable patients, 1 had a PR, 14 had SD for 2 to 16.3 months, and 6 progressed after T-cell infusion (118).

### c-Met

c-Met is a tyrosine kinase receptor encoded by MET proto-oncogene and can binds to HGF with high affinity (119). As previously described, c-Met exerts an important role in metastasis of HCC through c-Met/HGF signaling pathway. c-Met-targeting CAR-T cells have demonstrated anti-tumor efficacy in c-Met positive several malignancies such as renal carcinoma, gastric cancer and breast cancer (120–122). Huang and coworkers constructed the second and third generation of c-Met CAR-T and evaluated their anti-tumor abilities *in vitro* and *in vivo*. They confirmed that c-Met CAR-T cells could specifically lyse HCC cells with the third generation CAR-T cells displaying more potent anti-tumor capability *in vivo* (123). Additionally, to weaken the influence of HCC-suppressive tumor microenvironment on CAR-T, scientists tried to design a dual CAR directing c-Met and PD-L1. In comparison with c-Met CAR-T cells or PD-L1 CAR-T cells, this dual CAR-T showed increased anti-tumor ability against c-Met^+^ PD-L1^+^ HCC cells. Moreover, improved survival persistence was observed in these dual CAR-T cells (124).

### Alpha-fetoprotein (AFP)

AFP is a 70-KDa glycoprotein which is a well-established biomarker for HCC (125). In most HCC patients, AFP is detected at elevated levels and is associated with HCC progression and drug resistance (125, 126). Demonstrated by Liu et al., intratumoral administration of AFP CAR-T cells efficiently inhibited both HepG2 and AFP_158_-expressing tumors *in vivo*. Moreover, intravenous injection of AFP CAR-T cells suppressed tumor growth rapidly and profoundly in tumor-bearing mice. AFP CAR-T cells also showed potent antitumor activity in an established intraperitoneal HCC xenograft model (127).

### CD147

CD147, a transmembrane glycoprotein belonging to the immunoglobulin superfamily, is upregulated in kinds of malignancies, such as non-small cell lung cancer, breast cancer, and HCC (128, 129). Its involvement in the regulation of the TME and cancer progression, suggesting its potential as a promising target in cancers (129, 130). In Zhang’s research, a novel CAR-T cell system targeting CD147 induced by Doxycycline (Dox) was developed. The supply of Dox can be terminated immediately once severe adverse events occur, in which case the expression of CD147 CAR on T-cells will return to the baseline within 24-48 hours to minimize potential toxicities of CAR-T cells (131). Similarly, researchers also developed logic-gated (log) GPC3-synNotch-inducible CD147 CAR to minimize any on-target/off-tumor toxicity. LogCD147-CAR selectively lyses dual antigen (GPC3^+^CD147^+^), but not single antigen (GPC3^-^CD147^+^) positive cells and severe toxicity was not occurred in a human CD147 transgenic mouse model (132). Currently, a phase I clinical trial (NCT03993743) is ongoing to assess the clinical response of CD147 CAR-T in patients with advanced HCC.

### NK group 2 member D (NKG2D)

NKG2D, a type II transmembrane glycoprotein, is expressed on all NK cells, CD8^+^ T cells, some autoreactive CD4^+^ T cells and subsets of γδ T cells (133). Generally, NKG2D ligands (NKG2DL) are not detected on normal cells but exhibit elevated expression on tumor cells, suggesting potential targets for immunotherapy (134). In Sun’s study, the second generation human NKG2D CAR-T cells efficiently eliminated the NKG2DLs-expressing HCC cell *in vitro*, whereas they less efficiently killed NKG2DL-silenced or -negative cells; The subcutaneous xenograft model further illuminated that T cells expressing the NKG2D CAR effectively suppress tumor growth. Interestingly, NKG2D CAR-T cells derived from patients with HCC demonstrated anti-tumor ability and specifically eradicated NKG2DL-high HCC cells (134).

### Other promising targets

At present, various other potential targets for HCC are under investigated. MUC1 is a transmembrane glycoprotein, whose aberrant overexpression is identified on the surface of diverse human malignancies (135). Immunohistochemical analysis demonstrated that MUC1 was strong positive in 70.8% of liver cancer, while absent in normal liver tissues. Functionally, MUC1 participates in the migration and invasion by interacting with the HGF/c-Met and JNK/TGF-β signaling pathway and strongly correlates with metastasis and poor prognosis of HCC (136–138). Of note, MUC1 CAR-T exhibited antitumor potential against breast cancer (139, 140). At present, a basket trial of MUC1 CAR-T is underway in several malignancies including HCC (NCT02587689)

The melanoma antigen gene (MAGE) protein family consists of type I and II proteins (141). Normally, numerous MAGE proteins are only expressed in reproduction-related tissues, but aberrant expressions are observed in various tumors including HCC (141, 142). MAGE-1 and MAGE-3 mRNA expression is identified in 68% of HCC cases, but MAGE expression was no detected in the non-tumor samples (143). Wei’s search showed that overexpression of MAGE-A9 contributes to stemness and malignancy of HCC (144). To date, MAGE CAR-T cells for the treatment of lung cancer is underway in a phase I/II clinical trial (NCT03356808). Little information is available for MAGE CAR-T in HCC.

Epithelial cell adhesion molecule (EpCAM) is a transmembrane glycoprotein (145). Immunohistochemistry analysis revealed that EpCAM is broadly expressed by HCC and normal adjacent tissues; However, its expression is upregulated in tumor tissues and associated with poor prognosis in HCC patients (146). Functionally, EpCAM can maintain the capacity for malignant proliferation, invasion and metastasis (147–149). Several clinical trials to evaluate the EpCAM CAR-T cells for the treatment of advanced HCC are carrying out (NCT05028933, NCT03013712, NCT02729493).

As a foetal glycoprotein, carcinoembryonic antigen (CEA), is not usually expressed in significant quantity after birth but can be overexpressed on the cell surface of various cancers, such as colorectal, gastric, pancreatic, ovarian and lung cancer (150, 151). Under physiological conditions, the expression of CEA is restricted to the apical surface of epithelial cells towards the lumen to avoid recognition by immune cells (151). This unique expression pattern makes CEA an attractive target for immunotherapy. Currently, clinical trials using CEA CAR-T are mainly for the treatment of liver metastasis. A phase I/II basket trial to evaluate the efficacy and safety of CEA-targeted CAR-T cells is recruiting patients with relapse/refractory CEA^+^ tumors including liver cancer (NCT04348643).

Tumor endothelial marker 1 (TEM1) is the prototypical member of a family of genes expressed in the stroma of tumors, cancer cells and pericytes (152). In HCC, TEM1 is mainly expressed in CAFs and its expression inversely correlates with patient prognosis (153). TEM1 also contributes to the vascular adhesion, migration and invasion of tumor cells (105, 153). Julie et al. successfully constructed a second generation CAR-T to specifically target TEM1^+^ cells, confirming TEM1 as an attractive target for cancer immunotherapy (154).

New York esophageal squamous cell carcinoma (NY-ESO-1), a promising cancer testes antigen, is expressed by 43.9% of cases of HCC (155, 156). Very recently, NY-ESO-1 CAR-T cells constructed by Liu et al. suppressed tumor growth and prolonged the OS of mice in breast cancer and melanoma model (157).

## Challenges and strategies for CAR-T towards TME

**Figure 3:**
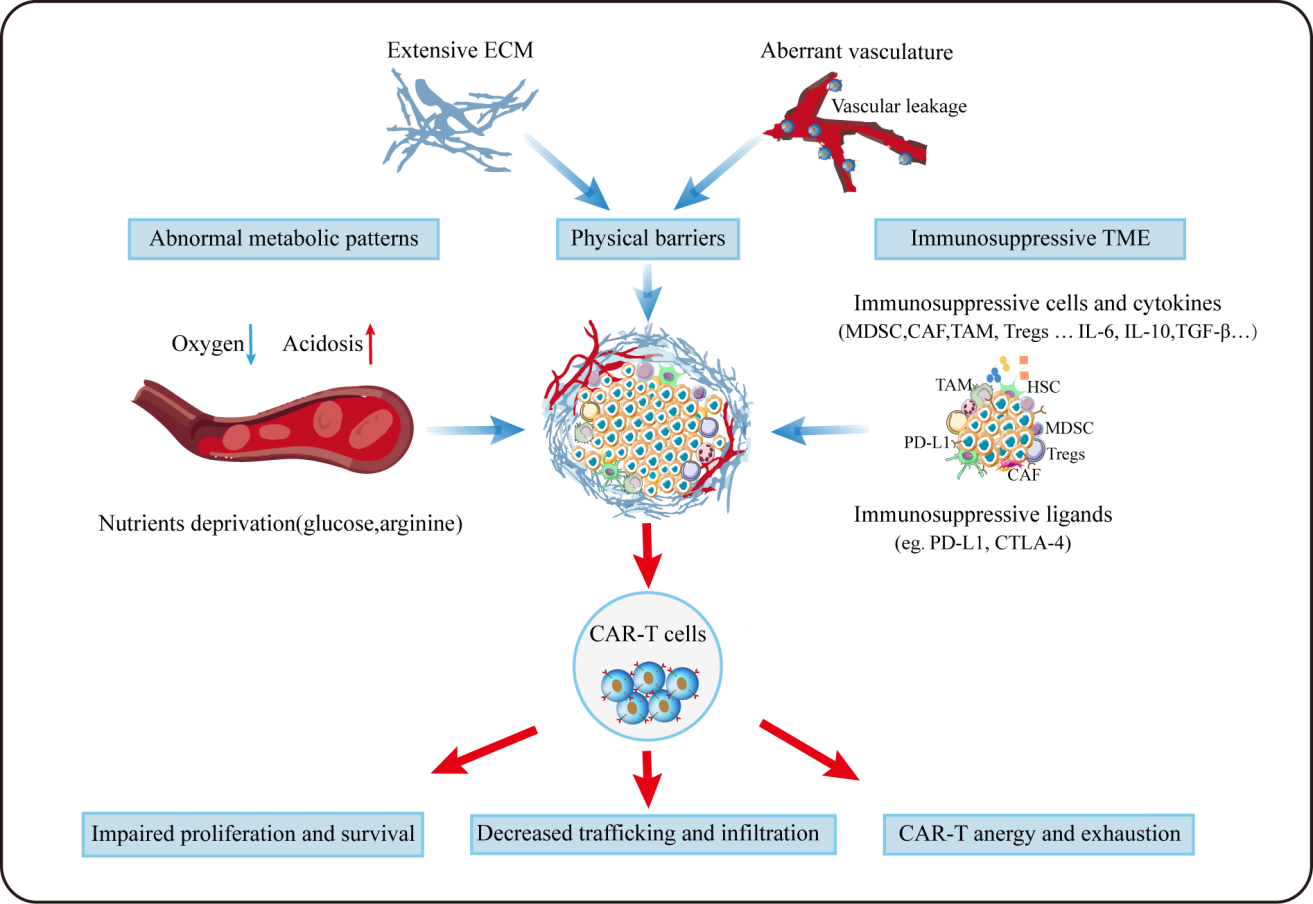
Challenges for CAR-T cells in TME. Aberrant vasculature and extensive ECM forms the special physical barriers making it difficult for CAR-T to efficiently traffic and infiltrate towards tumor tissues. Due to aberrant vasculature and the enhanced metabolism of tumor, CAR-T cells grow in a hypoxic, acidic and nutrition-deprivation milieu. Besides, immunosuppressive cellular and noncellular components can deactivate T cells *via* diverse mechanisms.

**Figure 4:**
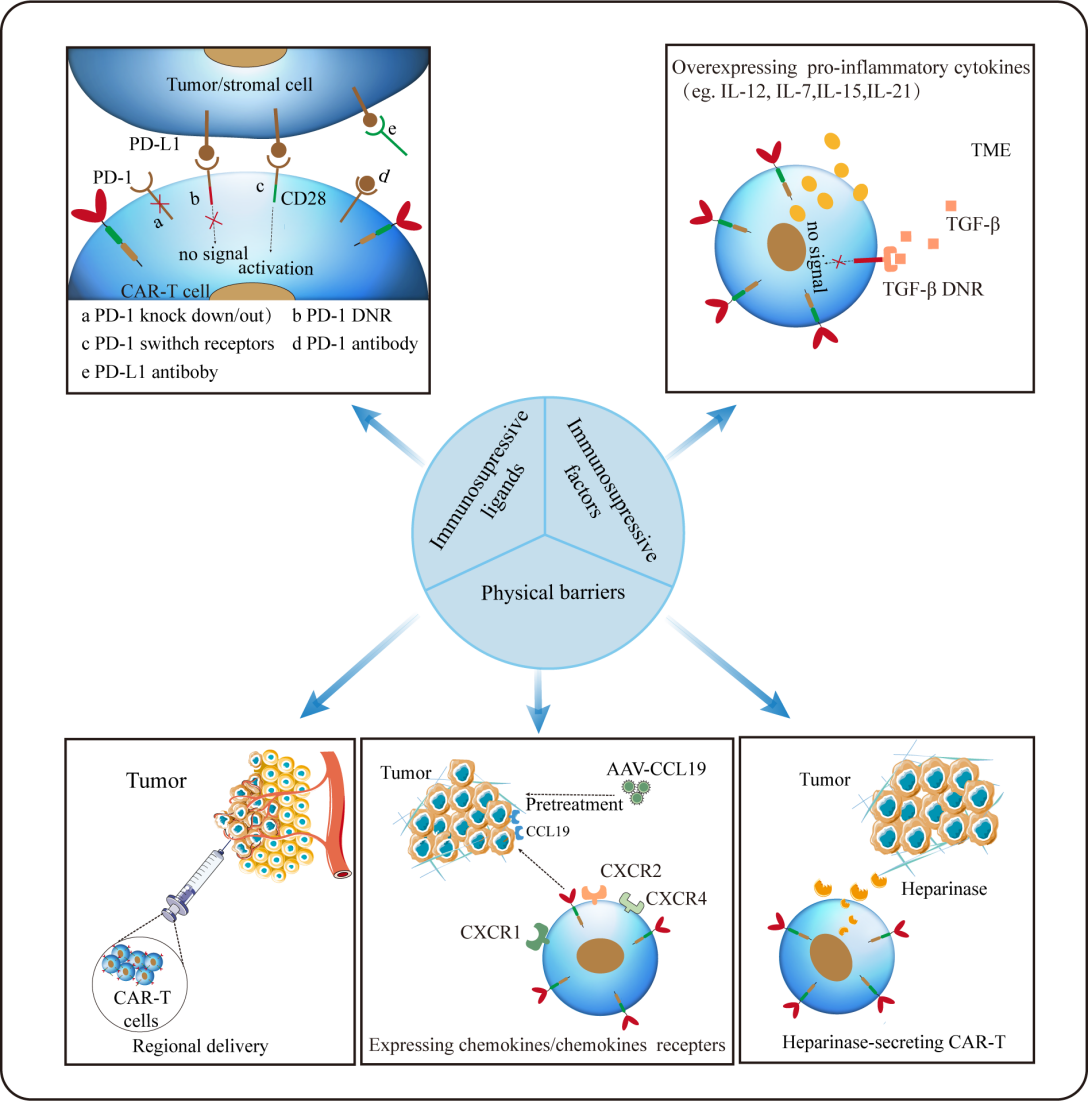
Strategies for CAR-T cells to overcome hostile TME. Regional delivery allows CAR-T cells to reach the tumor site directly. Inducing secretion of enzymes by CAR-T (eg. heparanase) to degrade ECM and optimizing CAR-T to express chemokines/chemokines receptors appears to remarkably improve CAR-T trafficking and infiltration. Disrupting the PD-1 expression on CAR-T cells or silencing/reversing the PD-1/PD-L1 axis can augment CAR-dependent antitumor activity. And modifying CAR-T to secrete pro-inflammatory factors may be an effective strategy against the inhibitory tumor microenvironment.

To date, the research of CAR-T therapy for HCC is in full swing around the world. Given its exceptional success in hematological malignancies, it may be very promising as a new approach for HCC treatment in future. But before that, there are still a series of difficulties remains to be overcome. In addition to tumor antigen heterogeneity and serious adverse events, the TME plays a non-negligible role in compromising the efficacy of CAR-T in HCC (**Figure 3**), and scientists are making much efforts to solve these problems (**Figure 4**).

Trafficking towards and infiltration into tumor tissue is a prerequisite for CAR-T cells to exert the anti-tumor function. Different from hematological malignancies, where CAR-T can directly target malignant cells, regarding to solid tumors, CAR-T need to traffic to the tumor lesions to bind to their target, which is often greatly limited by the hostile TME. On the one hand, special physical barriers such as abundant and aberrant neovascularization, wide gap of vessel walls, extensive vascular leakage and ECM make it difficult for CAR-T to efficiently go home to tumor tissues (158, 159). Obviously, HCC that develops from liver fibrosis and cirrhosis are highly fibrotic, which hamper CAR-T to traffic and infiltrate into tumor sites physically. On the other hand, solid malignancies often secret chemokines such as CXCL1, CXCL2 and CXCL5 to impede the migration and penetration of T cells (160, 161). Theoretically, regional delivery of CAR-T can compensate for poor trafficking while reducing systemic toxicity linked with intravenous administration (162). Notably, regional delivery of CAR-T cells to treat malignant pleural diseases has proven feasible, safe and demonstrated antitumor activity in a phase I trial (163). It was reported that inducing secretion of enzymes by CAR-T cells (eg. heparanase) to degrade ECM have resulted in improved infiltration (164). As aforementioned, the addition of CCL19 in CAR-T treatment improved CAR-T cells infiltration and survival in mice (114). Besides, optimizing CAR-T cells to express the corresponding receptors of chemokines derived from tumors appears to remarkably improve CAR-T cell trafficking. In HCC, CXCR2-expressing CAR-T cells significantly accelerate trafficking and accumulation in tumor, and exhibit improved anti-tumor efficacy (165). Overexpression of other chemokines receptors such as CXCR1 and CXCR4 also provide the advantage of penetration for therapeutic T cells (166, 167).

Unfortunately, even after migration into the tumor lesions successfully, it is still harsh for CAR-T cells to survive in a hostile milieu with various immunosuppressive factors (168). Firstly, TME is characterized by hypoxia, acidosis and nutrients deprivation resulting from the enhanced glycolytic metabolism of tumor cells (105). Secondly, as previously mentioned, immunosuppressive cellular components such as MDSCs, CAFs, TAMs and Tregs can deactivate T cells *via* diverse mechanisms including the production of tumor facilitating cytokines and growth factors. Thirdly, immune checkpoints like PD-1 and CTLA-4 in TME can act as suppressors to compromise antitumor immunity (168). Thus, combination checkpoint blockade with CAR-T cells is considered as the next immunotherapy modality. To date, much efforts to overcome these problems has been made. Christopher et al. constructed PSMA CAR-T cells which express a dominant-negative receptor (DNR) to block TGF-β signaling, and results showed that these T cells exhibit increased proliferation, cytokine release, decreased exhaustion and long-term persistence *in vivo* (169). Disrupting the PD-1 expression on CAR-T cells to evade PD-1/PD-L1 pathway has proven to augment CAR-dependent antitumor activity (170, 171). Given its improved efficacy in mesothelioma, combination checkpoints antibody with CAR-T after lymphodepletion may provide more possibilities for tumor immunotherapy (163). In addition, other investigations focusing on the pro-inflammatory cytokines instead of inhibitory signals have been carried out (172, 173). It was reported that inducible expression of IL-12 in CAR-T could boost antitumor activity in HCC (172).

## Conclusion

HCC is a highly heterogeneous malignant tumor. Its carcinogenesis and progression are the consequence of the interaction of multiple factors and mechanisms. The tumor microenvironment is an intricate network that plays a pivotal role in the evolution of HCC. Enhancing our knowledge of mechanisms of carcinogenesis and development in HCC will greatly benefit the exploration of novel therapeutic modalities. The success of CAR-T therapy in hematological malignancies demonstrates the potential of immunity and also bring the light of HCC immunotherapy, but more efforts are needed to improve its antitumor efficacy and safety before its widespread clinical application. Moreover, the role of TME in the treatment of HCC with CAR-T cells cannot be ignored. We are optimistic about that with the further in-depth study of cancer molecular biology and immunology, the treatment of HCC will finally usher in the dawn.

## Author contributions

YZ, SR, and RZ contributed to conception and design of the study. ZG and JG wrote the first draft of the manuscript. LL and WH wrote sections of the manuscript. All authors contributed to manuscript revision, read, and approved the submitted version

## Funding

This work was supported by National Natural Science Foundation of China (No. 82070643 and U1904164).

## Acknowledgments

We are grateful to National Natural Science Foundation of China for its support.

## Conflict of interest

The authors declare that the research was conducted in the absence of any commercial or financial relationships that could be construed as a potential conflict of interest.

## Publisher’s note

All claims expressed in this article are solely those of the authors and do not necessarily represent those of their affiliated organizations, or those of the publisher, the editors and the reviewers. Any product that may be evaluated in this article, or claim that may be made by its manufacturer, is not guaranteed or endorsed by the publisher.
